# Steigt die Prävalenz der rheumatoiden Arthritis wirklich an?

**DOI:** 10.1007/s00393-022-01192-7

**Published:** 2022-03-31

**Authors:** Katinka Albrecht, Johanna Callhoff, Anja Strangfeld

**Affiliations:** 1 Programmbereich Epidemiologie und Versorgungsforschung, Deutsches Rheuma-Forschungszentrum Berlin, Charitéplatz 1, 10117 Berlin, Deutschland; 2 Institut für Sozialmedizin, Epidemiologie und Gesundheitsökonomie, Charité Universitätsmedizin Berlin, Berlin, Deutschland; 3 Medizinische Klinik mit Schwerpunkt Rheumatologie und Klinische Immunologie, Charité Universitätsmedizin Berlin, Berlin, Deutschland

**Keywords:** Epidemiologie, Versorgungsforschung, Abrechnungsdaten, Sekundärdaten, Rheumatische Erkrankung, Epidemiology, Health Services Research, Claims analyses, Secondary data, Rheumatic disease

## Abstract

Immer mehr Auswertungen von Krankenkassendaten zeigen einen Anstieg der Prävalenz der rheumatoiden Arthritis (RA) in Deutschland. Die Studien beziehen sich auf die Abrechnungsdiagnose einer RA, die in Krankenkassendaten in den letzten Jahren im Vergleich zu früheren Zeiträumen häufiger zu finden ist. Je nach Falldefinition variieren die Zahlen zwischen 0,6% und 1,4% der erwachsenen Bevölkerung. In dieser Arbeit werden die verschiedenen Studien hinsichtlich der Datenquellen, der Falldefinitionen einer RA und der Diagnosehäufigkeit beleuchtet. Aufgrund der fehlenden klinischen Validierung lässt sich die Prävalenz anhand von Abrechnungsdaten nicht präzise bestimmen.

## Abstract (en)

A growing number of health insurance data analyses show an increase in the prevalence of rheumatoid arthritis (RA) in Germany. The studies refer to the claims diagnosis of RA, which is more frequent in recent years compared to earlier periods. Depending on the case definition, the numbers vary between 0.6% and 1.4% of the adult population. In this paper, the different studies are reviewed with regard to their data sources, the case definitions of RA and the frequency of the diagnosis. Due to the lack of clinical validation, the prevalence cannot be precisely determined from claims data.

Liest man die Nachrichten aus der Presse zu Rheumaerkrankungen, so häufen sich Berichte über einen Anstieg der Zahl von Personen mit rheumatischen Erkrankungen. Damit sind fast immer Personen mit rheumatoider Arthritis gemeint. „In Deutschland sind mehr Menschen an Rheuma erkrankt als gedacht“, formulierte das Ärzteblatt 2017 nach der Veröffentlichung von Daten aus dem Versorgungsatlas des Zentralinstituts für die kassenärztliche Versorgung (ZI) [1]. Im Jahr 2020 berichtet das Ärzteblatt „Die Zahl der Rheuma-Patienten steigt“ nach einer Auswertung von Versichertendaten der Kaufmännischen Krankenkasse (KKH) [2]. Bei gleicher Datenquelle titelte 2021 der Merkur „Rheuma-Schock: Dramatischer Anstieg in Bayern“ [3] und auch ein Artikel der Ostsee-Zeitung: „Immer mehr Rheuma-Patienten in MV“ [4] stützt sich auf die Krankenkassendaten der KKH. Vor dem Hintergrund der aktuellen Bedarfsplanung in der Rheumatologie, in der ermittelt wird, wie viele klinisch tätige Rheumatologen, rheumatologische Professuren und fachärztliche Ausbildungsstätten benötigt werden, um eine adäquate Versorgung zu gewährleisten, ist eine möglichst genaue Schätzung der Prävalenz entzündlich-rheumatischer Erkrankungen vonnöten. Was können diese Daten beitragen und wie sollten wir sie aus epidemiologischer und rheumatologischer Sicht hinsichtlich ihrer Versorgungsrelevanz interpretieren?

Die KKH hat bereits mehrfach in Pressemitteilungen Ergebnisse aus ihren Abrechnungsdaten vorgestellt und berichtet den prozentualen Anstieg an KKH-Versicherten mit rheumatoider Arthritis (RA) im Vergleich zu Daten von vor 10 Jahren, zuletzt bezogen auf 2009 und 2019. Hier wurde ein Anstieg um bundesweit 36 % mit dem höchsten Anstieg in Baden-Württemberg (+59 %) und in Sachsen (+54 %) berichtet [5]. In einem 2018 veröffentlichten Vergleich der Daten von 2007 und 2017 war die beschriebene Zunahme in Mecklenburg-Vorpommern mit 62 % am höchsten [6]. Die KKH hat ca. 1,6 Mio. Versicherte, entsprechend ca. 2 % der gesetzlich Krankenversicherten in Deutschland. Welche Abrechnungsdiagnosen für die Identifizierung einer RA verwendet wurden und wie viele Versicherte betroffen sind, wurde nicht angegeben.

Die Zahlen der KKH und auch des ZI beruhen auf Abrechnungsdiagnosen, die im Rahmen der vertragsärztlichen Abrechnung dokumentiert werden. Die Diagnose einer rheumatoiden Arthritis (RA) kann über die ICD-10 (Internationale statistische Klassifikation der Krankheiten, 10. Revision) als M05 (seropositive chronische Polyarthritis) oder M06 (sonstige chronische Polyarthritis, unter die auch die seronegative RA fällt), codiert werden. In vielen Auswertungen von Abrechnungsdaten werden alle mit M05 und M06 verschlüsselten Diagnosen als RA eingeordnet.

Das ZI verwendet als Datenquelle die bundesweiten vertragsärztlichen Abrechnungsdaten der gesetzlichen Krankenversicherungen. Diese umfassen alle gesetzlich Krankenversicherten (ca. 70 Mio.), entsprechend fast 90 % der Bevölkerung. In ihrer Auswertung von 2017 wurde eine RA angenommen, wenn eine ICD-10-Codierung (M05, M06) in mindestens 2 von 4 aufeinanderfolgenden Quartalen vorlag und zusätzlich ein Entzündungsmarker (C-reaktives Protein oder Blutsenkungsgeschwindigkeit) in einem der Quartale untersucht wurde. Die Prävalenz wurde als prozentualer Anteil der Versicherten mit RA-Diagnose an der Gesamtpopulation berechnet. Laut dieser Daten stieg die Häufigkeit der RA zwischen 2009 und 2015 von 0,9 % (*n* = 526.211) auf 1,1 % (*n* = 666.220) an [7].

In einer weiteren Auswertung von 2021 berichtet das ZI einen kontinuierlichen Anstieg der Diagnoseprävalenz zwischen 2012 und 2018, dies betrifft neben der RA auch andere Autoimmunerkrankungen – ausgewertet wurden Abrechnungsdiagnosen zu M. Crohn, Colitis ulcerosa, Psoriasis und multiple Sklerose. Die Prävalenz der RA-Diagnose stieg in diesem Zeitraum von 1,2 auf 1,4 %, allerdings war in dieser Auswertung auch die juvenile Arthritis (M08) als Diagnose eingeschlossen, und es wurde keine Entzündungsdiagnostik vorausgesetzt [8]. Daher sind die Zahlen nicht direkt mit der früheren Auswertung vergleichbar.

Im Rahmen der Versorgungsforschung ist es durchaus von Interesse, Abrechnungsdaten auch für epidemiologische Fragestellungen zu nutzen – sie haben gegenüber Auswertungen von Beobachtungsstudien, Registern und regionalen Erhebungen den Vorteil, dass sie bevölkerungsbezogene Analysen ermöglichen und (abgesehen von den gut 10 % Privatversicherten) alle Personen, weitgehend unabhängig von Alter, Geschlecht, sozioökonomischem Status und fachärztlichen Kontakten einschließen.

Bei Aussagen zur Prävalenz muss aber immer klar formuliert sein, dass es sich bei Krankenkassendaten um keine klinisch validierten Diagnosen handelt. Es gibt keine spezifischen Informationen zur Diagnosestellung, Symptomdauer, dem Gelenkbefall, der Serologie und der Höhe der Entzündungsmarker. Abrechnungsdiagnosen können fehlerhaft codiert, bei Änderung der Diagnose nicht korrigiert oder ungefragt übernommen worden sein.

Um die Limitation der fehlenden klinischen Validierung zu berücksichtigen, war in der PROCLAIR-Studie 2013 zunächst die Diagnoseprävalenz der RA (M05, M06) in Abrechnungsdaten der BARMER mit verschiedenen Falldefinitionen angenähert worden. Die BARMER ist mit ca. 8,7 Mio. Versicherten (entsprechend ca. 12 % der gesetzlich Krankenversicherten) derzeit die zweitgrößte gesetzliche Krankenversicherung in Deutschland. Je nachdem, ob nur die Diagnose (in mindestens zwei Quartalen), eine Bestimmung der Entzündungsmarker, zusätzlich eine antirheumatische Medikation oder sogar ein rheumatologischer Kontakt vorausgesetzt wurde, lag die auf die deutsche Bevölkerung standardisierte Prävalenz im Jahr 2013 zwischen 1,4 und 0,6 % [9]. Während die allein durch die Abrechnungsdiagnose definierte RA die Prävalenz sicher überschätzt, da sie auch alle fehlerhaften Codierungen einschließt, wird die Prävalenz bei der Voraussetzung eines rheumatologischen Kontaktes sicher unterschätzt, weil alle Personen, die ausschließlich hausärztlich oder anderweitig fachärztlich versorgt werden, ausgeschlossen sind. Am überzeugendsten und im Einklang mit der bisher für Deutschland geschätzten Prävalenz von 0,8 % [10] erscheint die Definition der Diagnoseprävalenz, wenn zusätzlich zu der Abrechnungsdiagnose eine Kontrolle der Entzündungsmarker (1,0 %) bzw. eine zusätzlich bestehende entsprechende Medikation (0,8 %) vorlag. Hierzu zählten konventionell synthetische „disease-modifying antirheumatic drugs“ (csDMARDs), biologische DMARDs, aber auch Glukokortikoide und NSAR, wohl wissend, dass NSAR nicht zur Abgrenzung gegenüber nichtentzündlichen Diagnosen geeignet sind. Schließt man sie aber aus, werden alle Personen, die nur mit NSAR behandelt werden, nicht berücksichtigt – was häufiger in der nicht fachärztlichen Versorgung, aber auch bei rheumatologisch betreuten Patienten vorkommt [11, 12].

Das ZI hat in seiner Analyse von 2017 die zweite Falldefinition (Diagnose plus Abrechnung einer Laboruntersuchung der Entzündungsmarker) verwendet und damit eine gute Rationale für die Annäherung an die tatsächliche Prävalenz gefunden.

Auch andere Auswertungen von Abrechnungsdaten verfolgten den Ansatz der engeren Falldefinitionen. Eine Studie mit Daten der AOK Baden-Württemberg (4,5 Mio. Versicherte) ergab eine Prävalenz der RA-Diagnose (M05, M06) je nach Definition zwischen 1,1 % (nur Diagnose) und 0,6 % (Diagnose und spezifische Medikation) [13].

In einer Studie mit Daten aus der Forschungsdatenbank der Vilua Healthcare GmbH (Daten von 7 gesetzlichen Krankenversicherungen, ca. 3,5 Mio. Versicherte) wurde 2008–2013 eine Prävalenz der RA-Diagnose (M05, M06) von 1,3 % insgesamt und bei fachärztlicher Diagnosestellung von 1,0 % berichtet [14]. Eine weitere Analyse aus der gleichen Datenquelle mit einer engeren Diagnosedefinition (es wurden nur M05.8, M06.0 und M06.8 eingeschlossen) ergab eine Prävalenz von 0,4–0,5 % zwischen 2012 und 2016 [15]. Damit wurden aber die am häufigsten verwendeten unspezifischen Codierungen der RA (M05.9, M06.9) nicht berücksichtigt, was sicher zu einer deutlichen Unterschätzung geführt hat.

Im Gegensatz zu der ausschließlichen Analyse codierter Abrechnungsdiagnosen wurde in der PROCLAIR-Studie in einem zusätzlichen Schritt eine zufällige, nach Abrechnungsdiagnose (M05/M06), Geschlecht und Alter stratifizierte Stichprobe von 6600 Versicherten angeschrieben und befragt, ob die Versicherten die Diagnose bestätigen konnten. Es haben 51 % auf das Anschreiben geantwortet. Hiervon bestätigten 81 % die RA-Diagnose [11]. Von den 19 %, die die Diagnose nicht bestätigten, hatte die Mehrzahl eine andere entzündlich-rheumatische Diagnose (am häufigsten Psoriasisarthritis oder axiale Spondyloarthritis) oder eine Arthrose. Bei seropositiv codierter RA (M05) wurde die Diagnose mit 94 % deutlich häufiger bestätigt als bei sonstiger RA (M06) mit 76 %. Nimmt man zusätzliche Kriterien hinzu, dann ist die spezifische Medikation das Einzelkriterium mit dem höchsten positiven prädiktiven Wert. Die Analyse zeigt, dass ICD-10-Codes zur Selektion von Versicherten mit einer RA zwar gut geeignet sind, sie verdeutlicht aber auch den Unsicherheitsbereich, der bei der Nutzung von Abrechnungsdiagnosen beachtet werden muss. Ein wichtiger Aspekt ist, dass diese Unsicherheit durch die Anwendung weiterer Auswahlkriterien reduziert und ein genauerer Näherungswert erzielt werden kann.

Einen alternativen Ansatz zu Auswertungen von Krankenkassendaten liefert die NAKO Gesundheitsstudie. Diese erfüllt formal die Kriterien eines bevölkerungsbezogenen Surveys. Für die Befragung wurden von 2014 bis 2019 über 200.000 Frauen und Männer im Alter von 20–69 Jahren aus Zufallsstichproben der Allgemeinbevölkerung in 18 Studienzentren rekrutiert [16]. Im Rahmen der Angaben zu seltenen Erkrankungen wurden die Teilnehmer:innen u. a. befragt, ob sie eine ärztlich diagnostizierte RA/Polyarthritis haben. Von den ersten 100.000 Teilnehmer:innen bestätigten dies 1,85 % (standardisiert auf die deutsche Bevölkerung) [17]. Eine Überschätzung der Diagnose ist bei einer reinen Befragung durch die leichte Verwechslung von RA und Fingerpolyarthrosen („Rheuma“) seitens der Betroffenen anzunehmen. Abgeschwächt wird die Interpretation der Daten auch durch die insgesamt niedrige Antwortrate von 18 % und durch die eingeschränkten Altersgrenzen. Daher sollten die Daten der NAKO Gesundheitsstudie nicht für Prävalenzberechnungen herangezogen werden.

Mögliche Gründe für einen tatsächlichen Anstieg der RA-Prävalenz in Deutschland sind zum ersten eine Veränderung in der Altersstruktur der Bevölkerung: Laut Daten des Statistischen Bundesamtes stieg in Deutschland die Zahl der Menschen im Alter ab 67 Jahren zwischen 1990 und 2018 von 10,4 auf 15,9 Mio. Die Zahl der über 80-Jährigen stieg zwischen 1970 und 2017 von 1,2 auf 5,2 Mio., damit erhöhte sich ihr Anteil an der Gesamtbevölkerung von 1,9 auf 6,2 % [18]. Dies führt zwangsläufig zu einem deutlichen Anstieg älterer Menschen mit RA und anderen entzündlich-rheumatischen Diagnosen, der sich auch in den Abrechnungsdaten widerspiegelt. In den BARMER-Daten hat sich die Zahl der Versicherten mit einer RA-Diagnose zwischen 2005 (*n* ≈ 79.000) und 2020 (*n* ≈ 145.000) nahezu verdoppelt, der Anteil über 80-Jähriger stieg im gleichen Zeitraum von 13 auf 23 %. Gleichzeitig ist die Mortalität der RA in den letzten Jahrzehnten in vielen Ländern und auch in Europa gesunken, welches auf die verbesserte Behandlung der RA und ihrer Komorbiditäten bei niedrigerer Krankheitsaktivität zurückgeführt wird [19, 20]. Neben der RA wird auch eine Zunahme anderer Autoimmunerkrankungen wie multiple Sklerose oder Typ-1-Diabetes in der Bevölkerung beobachtet. Bei stabilen Inzidenzraten wird dies bei einer gestiegenen Lebenserwartung am ehesten auf einen Anstieg an prävalenten Fällen durch das spätere Versterben zurückgeführt [8].

Andere mögliche Gründe für einen Anstieg der RA-Diagnosen in Abrechnungsdaten sind die Zunahme an sensitiverer Diagnostik (z. B. MRT, Frühsprechstunden), Veränderungen im Codierverhalten (Möglichkeit der Abrechnung spezieller Diagnostik, Medikamente oder Heilmittel bei entsprechender ICD-Diagnose) und eine gestiegene Inanspruchnahme der vertragsärztlichen Versorgung.

Um die tatsächliche Prävalenz der RA und anderer entzündlich-rheumatischer Erkrankungen sicher zu ermitteln, bedarf es mindestens zweistufiger Bevölkerungsstudien, die auch eine klinische Diagnostik umfassen. Aufgrund ihrer Komplexität kann diese Art von Studien nur selten und auch nur regional begrenzt durchgeführt werden. Die einzige deutsche Bevölkerungsstudie bleibt bislang die von Wasmus, Raspe et al., die 1989 in der Region Hannover durchgeführt wurde [21] und im Einklang mit anderen internationalen Bevölkerungsstudien [10] eine Prävalenz der RA je nach Definition von 0,6 bis 0,9 % ergab. Bei dieser Studie hatten 87 % der angeschriebenen Einwohner Hannovers den Fragebogen zu verschiedenen rheumatischen Beschwerden zurückgeschickt. Personen mit rheumatischen Beschwerden wurden anschließend rheumatologisch untersucht. Seit der Einführung der ACR/EULAR-Kriterien von 2010 gibt es keine neueren Studien, die die frühere Diagnosestellung einer RA im Vergleich zu älteren Daten berücksichtigt. Es ist also allein durch die Frühdiagnose heute eine etwas höhere Prävalenz anzunehmen.

## Fazit

Verschiedene Auswertungen von Abrechnungsdaten zeigen, dass die Zahl der Versicherten mit einer RA-Diagnose und auch mit anderen entzündlich-rheumatischen Erkrankungen in den letzten Jahren deutlich angestiegen ist. Durch eine immer älter werdende Bevölkerung in Kombination mit verbesserten Therapieoptionen der RA gibt es gute Gründe, einen tatsächlichen Anstieg der Anzahl von Betroffenen mit entzündlich-rheumatischen Erkrankungen in der Bevölkerung anzunehmen. Die genaue Anzahl an RA-Erkrankten in Deutschland kann und sollte aber nicht aus Abrechnungsdaten allein abgeleitet werden, denn letztlich weisen alle vorgestellten Studien die Unzulänglichkeit der fehlenden klinisch validierten Diagnose auf. Ein genauer Blick auf die zugrunde liegenden Datenquellen und die gewählte Definition der RA-Diagnose sowie ein bewusster Umgang mit dem Begriff Prävalenz ist für die Interpretation der Studien unabdingbar, bevor belastbare Aussagen getroffen werden können. Basierend auf allen verfügbaren Daten schätzen wir, dass die Prävalenz der RA in der erwachsenen Bevölkerung heute in einem Bereich zwischen 0,8% und 1,2% liegt. 
